# Age-related changes in the ratio of Type I/III collagen and fibril diameter in mouse skin

**DOI:** 10.1093/rb/rbac110

**Published:** 2022-12-29

**Authors:** Jianping Gao, Zhenhu Guo, Yang Zhang, Yuying Liu, Fangyu Xing, Junjie Wang, Xi Luo, Yingjun Kong, Guifeng Zhang

**Affiliations:** National Key Laboratory of Biochemical Engineering, Institute of Process Engineering, Chinese Academy of Sciences, Beijing 100190, China; School of Chemical and Engineering, University of Chinese Academy of Sciences, Beijing 100049, China; National Key Laboratory of Biochemical Engineering, Institute of Process Engineering, Chinese Academy of Sciences, Beijing 100190, China; School of Chemical and Engineering, University of Chinese Academy of Sciences, Beijing 100049, China; National Key Laboratory of Biochemical Engineering, Institute of Process Engineering, Chinese Academy of Sciences, Beijing 100190, China; National Key Laboratory of Biochemical Engineering, Institute of Process Engineering, Chinese Academy of Sciences, Beijing 100190, China; School of Chemical and Engineering, University of Chinese Academy of Sciences, Beijing 100049, China; National Key Laboratory of Biochemical Engineering, Institute of Process Engineering, Chinese Academy of Sciences, Beijing 100190, China; National Key Laboratory of Biochemical Engineering, Institute of Process Engineering, Chinese Academy of Sciences, Beijing 100190, China; National Key Laboratory of Biochemical Engineering, Institute of Process Engineering, Chinese Academy of Sciences, Beijing 100190, China; School of Chemical and Engineering, University of Chinese Academy of Sciences, Beijing 100049, China; National Key Laboratory of Biochemical Engineering, Institute of Process Engineering, Chinese Academy of Sciences, Beijing 100190, China; School of Chemical and Engineering, University of Chinese Academy of Sciences, Beijing 100049, China; National Key Laboratory of Biochemical Engineering, Institute of Process Engineering, Chinese Academy of Sciences, Beijing 100190, China; School of Chemical and Engineering, University of Chinese Academy of Sciences, Beijing 100049, China

**Keywords:** collagen, skin, ratio, fibril diameter

## Abstract

The content of type I collagen (COL-I) and type III collagen (COL-III) and the ratio between them not only affect the skin elasticity and mechanical strength, but also determine the fibril diameter. In this research, we investigated the age-related changes in COL-I/COL-III ratio with their formed fibril diameter. The experimental result was obtained from high performance liquid chromatography–mass spectrometer, hydroxyproline determination, picrosirius red staining and transmission electron microscopes (TEM), respectively. The result indicated that the COL-I/COL-III ratio in mouse skin increased with aging. From the 0th to 9th week, the COL-I/COLIII ratio increased from 1.3:1 to 4.5:1. From the 9th to the 18th week, it remained between 4.5:1 and 4.9:1. The total content of COL-I and COL-III firstly increased and then decreased with aging. The TEM result showed that the fibril diameter increased with aging. From the 0th to 9th week, the average fibril diameter increased from 40 to 112 nm; From the 9th to 18th weeks, it increased from 112 to 140 nm. After the 9th week. The fibril diameter showed obvious uneven distribution. Thus, the COL-I/COLIII ratio was proportional to the fibril diameter, but inversely proportional to the uniformity of fibril diameter.

## Graphical abstract

**Figure:**
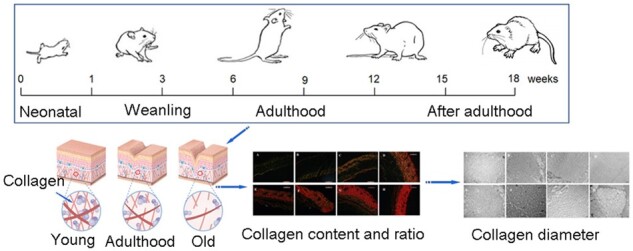

## Introduction

Collagen, which is the most widespread extracellular matrix protein, can be served as a significant ingredient for maintaining the cellular microenvironment and providing various functionality such as cell adhesion, cell migration, tissue morphogenesis and repair [1, 2]. Collagen makes up 25–35% of the total protein and provides the overall tissue stiffness and integrity in mammals [3, 4]. As the most abundant and significant collagen proteins, type I collagen (COL-I) and type III collagen (COL-III) widely exist in skin tissue, especially in dermis [5–7]. COL-I belongs to the coarse fiber and is usually regarded as structural scaffold to maintain the mechanism strength of skin. Additionally, it is an important component of reticular fiber in the interstitial tissue of the skin [5, 8]. COL-III, which belonging to tiny fiber, usually exists in parallel with COL-I to provide the elasticity for skin [9]. Moreover, it could combine with COL-I *in vivo* to form collagen fibrils [10, 11], a basic building block in the complex hierarchical structure of collagenous tissues [12–14].

Recent researches revealed that the content and ratio of COL-I and COL-III can represent the occurrence and development of diseases [15]. For example, the characterization of Vascular Ehlers–Danlos syndrome is the reduction of COL-III in the skin and vascular [16]. The number of collagen bundles in patients of amyotrophic lateral sclerosis (ALS) is decreased. Meanwhile, the diameter of the skin collagen fibrils increased with the duration of ALS development [17, 18]. The COL-I/COL-III ratio significantly decreased in the skin of patients with hernia [19]. Additionally, the COL-I/COL-III ratio in scar tissues was about 5:1, and the diameter of the collagen fibrils was much larger [20]. In addition to the disease, the content of COL-I and COL-III and their ratio change with the increase of age, affecting the skin properties such as strength and elasticity [21–24]. For instance, previous researches investigated that embryonic dermis contains about 50% COL-III, but it reduces to about 15% during post-natal growth [25, 26]. However, the studies about the COL-I/COL-III ratio and collagen fibril diameter with aging were rarely reported.

Therefore, in this study, the age-rated dynamic change of COL-I/COL-III ratio and fibril diameter was systematically investigated by a high-performance liquid chromatography-tandem mass spectrometer (HPLC–MS) analysis and morphology observation, respectively. The collagen was firstly digested by trypsin to identify the marker peptides for quantitively analyzing the content of COL-I and COL-III with HPLC–MS. Afterwards, the total content of COL-I and COL-III, and the ratio between them were investigated by the hydroxyproline method and picrosirius red staining, respectively. Additionally, the collagen fibril diameter of the mouse skin was characterized by transmission electron microscopes (TEM). The obtained results performed that the COL-I/COL-III ratio and the diameter of collagen fibril were both increasing with aging. The diameter of collagen fibril was proportionally to the COL-I/COL-III ratio.

## Materials and methods

### Materials

Mouse (C57BL/6N, male) were purchased from Beijing Charles River Laboratories. Chromatographic pure acetonitrile (Merck, USA), Chromatographic pure formic acid (Merck), Sequence-grade trypsin (Promega, USA), Two marker peptides (GSEGPQGVR and GPSGFR) and internal peptide (GLAGMK) were obtained from China Peptides Co., Ltd. The HPLC purity was 99.10% and 99.05% for GSEGPQGVR and GPSGFR, respectively.

### Preparation of collagen peptides

Animals’ experiments were resolved with guidance from Laboratory Animal Research Center of Tsinghua University. The mice (*n* = 6) were dislocated and killed at designed time intervals (0, 1, 3, 6, 9, 12, 15 and 18 weeks) to obtain back skin tissue, which was then mixed with the degreasing solution in the mass ratio of 1:5 to remove the fat. The degreasing solution consisted of trichloromethane and methyl alcohol (v/v, 2:1). After 24 h, the solvent was removed and replaced by fresh degreasing solution for two times. Until the organic solvents were dried, the prepared acetic acid solution (0.05 mol/l) was mixed with the above sample in a mass ratio of 1:2, which was then put in 4°C for 12 h. Finally, the mixture was swelled, homogenized, freeze dried and weighted.

Next, the dried sample (5 mg) was added to 5 ml NH_4_HCO_3_ (0.05 M, pH 8.0) solution. The mixture was degenerated at 60°C for 30 min. Then it was cooled to room temperature and mixed with 100 μl trypsin solution (0.2 mg/ml). The mixture was incubated at 37°C for 18 h. The enzymatic hydrolysate was centrifuged at 12 000×g for 10 min, and the supernatant was collected. Finally, the digested collagen peptides were obtained.

### Preparation of marker peptides and collagen peptide solution

The mixed standard marker peptides of COL-I and COL-III with different concentrations were prepared with NH_4_HCO_3_ (0.05 mol/l, pH 8.0) solution. Meanwhile, the internal standard peptide (GLAGMK) solution was also prepared with the same method. The internal standard peptide solution was combined with the mixed solution of COL-I and COL-III marker peptides and the sample enzymolysis solution at a ratio of 1:1 (V/V), respectively. Then they were centrifuged at 12 000×g for 10 min. The supernatant was collected for analysis.

### Quantification of hydroxyproline by HPLC

The samples were hydrolyzed with 6 M HCl at 110°C for 16 h. Subsequently, the liberated amino acids were derived with DNFB [27–29]. Finally, the solution was centrifuged at 12 000×g for 10 min to obtain supernatant for analysis.

The contents of hydroxyproline (Hyp) in mouse skin were detected as reported [29–31]. The liberated amino acids were analyzed through HPLC on Zorbax SB-C_18_ column (4.6 mm × 250 mm, 5 μm) (Agilent, USA).

### Picrosirius red staining

The samples were fixed in 10% formalin for 24 h, then embedded in paraffin. Then 5 μm sessions were taken and de-wax with xylene. After that, the slides were washed with tap water and stained with 0.1% fast green FCF for 10 min followed by acetic acid washing. The slides were then stained with picrosirius red F3BA for 1 h. The slides were subsequently washed with acidified water, dehydrated, cleared and mounted [24, 30, 31]. Finally, the slides were collected for polarized light microscopy analysis.

The slides stained with Picrosirius red were examined under polarized light microscopy with a microscope (DMi8, Leica, Germany) with a 10× objective. The images were processed by IPWIN32 software. The COL-I/COL-III ratio was calculated.

### TEM observation

The back skin was cut into 1 cm^2^ pieces, and washed with PBS (0.1 mol/l, pH 7.4) for three times. Then they were fixed in 1% osmic acid (0.1 mol/l PBS, pH 7.4) and washed with PBS (0.1 mol/l, pH 7.4) for three times, 15 min for every time. Samples were dehydrated in ethanol with stepwise concentrations of 0%, 10%, 20%, 30%, 50%, 70%, 90% and 100% (v/v) for 15 min. They were washed with acetone for two times and incubated with acetone: epoxy resin (v/v, 1:1) at 37°C for 3 h, acetone:epoxy resin (v/v, 1:2) at 37°C for 12 h, epoxy resin at 37°C for 8 h, respectively. The epoxy resin was loaded into the embedding plate. The samples were inserted into the embedding plate and baked overnight at 37°C. The embedding plate was incubated at 60°C for 48 h. Ultrathin sections (60–80 nm) were cut with a diamond knife on an ultramicrotome [Leica, UC17, Germany]. The sections were stained with 2% uranium acetate saturated alcohol solution and 2.6% lead citrate. Then they were washed with ultrapure water for three times [32, 33]. The slides were collected for TEM (JEM-1400Flash, JEOL, Japan) analysis. The fibril diameter was analyzed by Image J software.

### Marker peptides identification by HPLC–MS

The marker peptides of COL-I and COL-III were identified by HPLC/MS. The on-line chromatographic separation was performed by reversed-phased chromatography on a Peptide BEH C_18_ column (2.1 × 150 mm, 1.7 μm) (Waters, USA) by using the UHPLC (Vanquish, Thermo Fisher, USA). The mobile phase consisted of water with 0.1% formic acid (A) and 60% acetonitrile with 0.1% formic acid (B). The gradient elution procedure was performed below: 0–60 min, 5–40% B; 60–85 min, 40–90% B; 85–98 min, 90% B; 98–100 min, 90–5% B; 100–110 min, 5% B. The flow rate was 0.2 ml/min, the injection volume was 10 μl, and the column temperature was held at 60°C. The outlet of the column was introduced into an orbitrap mass spectrometer (Exploris 480, Thermo Fisher). Electron spray ionization (ESI) positive mode was used to perform the orbitrap mass spectrometry. The spray voltage was set to 3.5 kV. The capillary temperature and vaporizer temperature were 320°C and 300°C, respectively. The sheath gas was 19.8 ml/min. The aux gas was 5 psi. The MS scan range was set from m/z 300 to 2000, and the resolution was set to 60 000. The RF lens was 45%. The normalized AGC Target was 300%. The maximum ion injection time was 100 ms. The scan event 2 was data-dependent MS/MS and the resolution was set to 15 000. The isolation Window was m/z 1.6. The maximum ion injection time was 200 ms and the normalized AGC Target was 100%. The MS/MS collision energy was 30%. The mouse COL-I and COL-III maker peptides were identified through the SEQUEST algorithm in Protein Discoverer 2.4 software (Thermo Fisher).

### Collagen quantification by HPLC–MS

The marker peptides and digested samples were separated with a Zorbax C_18_ column (2.1 × 150 mm, 5 μm) (Agilent, USA) using an HPLC (U3000, Thermo Fisher). The mobile phase consisted of water with 0.1% formic acid (A) and 60% acetonitrile with 0.1% formic acid (B). The gradient elution procedure was performed below: 0–3 min, 5–25% B; 3–7 min, 25–100% B; 7–8 min, 100% B; 8–8.1 min, 100–5% B; 8.1–10 min, 5% B. The flow rate was 0.2 ml/min, the injection volume was 5 μl, and the column temperature was held at 30°C. The outlet of the column was introduced into TSQ mass spectrometer (Quantumn Access MAX, Thermo Fisher). The ESI positive mode was used to perform the orbitrap mass spectrometry. The spray voltage was set to 3.5 kV. The capillary temperature and vaporizer temperature were 320°C and 300°C, respectively. The sheath gas was 19.8 ml/min. The aux gas was 5 psi. The selected reaction monitoring was used. The monitored target ions of COL-I and COL-III were m/z 443.76 → 613.29 (GSEGPQGVR) and m/z 310.67 → 466.34 (GPSGFR), respectively. The monitored target ion of the internal peptide was m/z 288.66 → 406.18 (GLAGMK).

The contents of COL-I and COL-III in the sample were calculated by

$$X_{i}=\frac{C_{i}\times X\times V\times M_{1}}{m\times M_{2}\times n\times 1000}$$

*X_i_* is the content of COL-I or COL-III in the sample, mg/mg; *C_i_* is the concentration of COL-I or COL-III marker peptides, µg/ml; *V* is the final volume of the sample, ml; *M*_1_ is the molecular weight of COL-I or COL-III, Da; *M*_2_ is the molecular weight of COL-I or COL-III marker peptide, Da; m is the final sample weight, mg; *X* is the dilution ratio of the hydrolyzed sample; *n* is the conversion coefficient of COL-I or COL-III, which was listed in Table 1.

**Table 1.** The conversion coefficient of type I and type III collagen

Collagen type	Peptide chain	Conversion coefficient (*n*)
Type I	α1 chain	2
Type I	α2 chain	1
Type III	α1 chain	3

### Statistical analysis

All of the quantitative data were presented as mean ± standard deviation. Statistical analyses were carried out by the *t*-test. The *P*-values <0.05 were considered statistically significant.

## Results

### Detection of COL-I and COL-III in mouse skin

**Figure 1.**
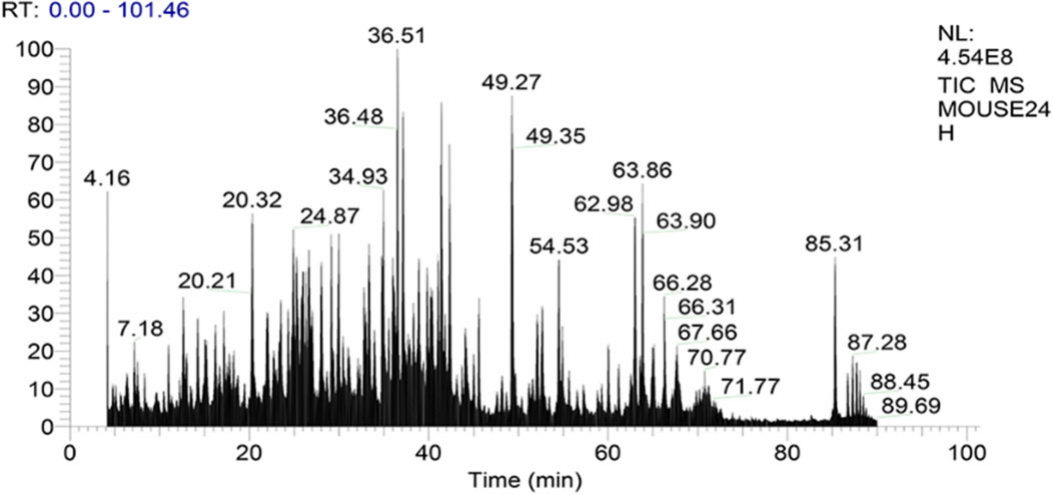
Total ions mass spectrum of mouse collagen digestion. The skin tissue was dissolved with NH_4_HCO_3_ (0.05 M, pH 8.0) solution, degenerated at 60°C for 30 min, and digested by trypsin at 37°C for 18 h.

**Figure 2.**
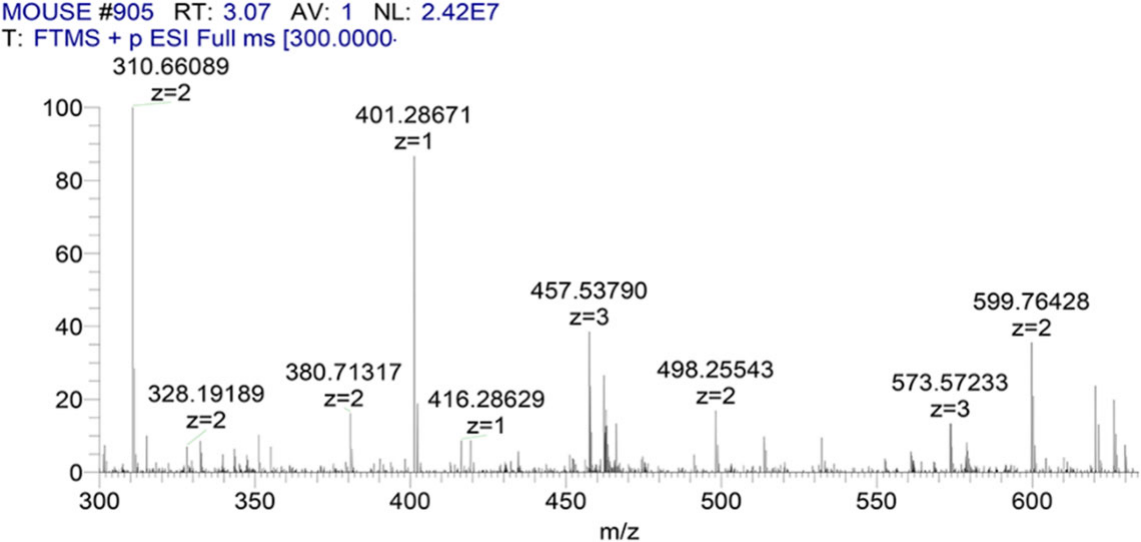
Mass spectrum of m/z 310.6609 detected in digested mouse skin. Based on the analysis of the difference in m/z of the isotope peaks, the ion of m/z 310.6609 was obtained with two charges. Therefore, the molecular weight of m/z 310.6609 was 619.307, which was consistent with the molecular weight of peptide (GPSGFR).

**Figure 3.**
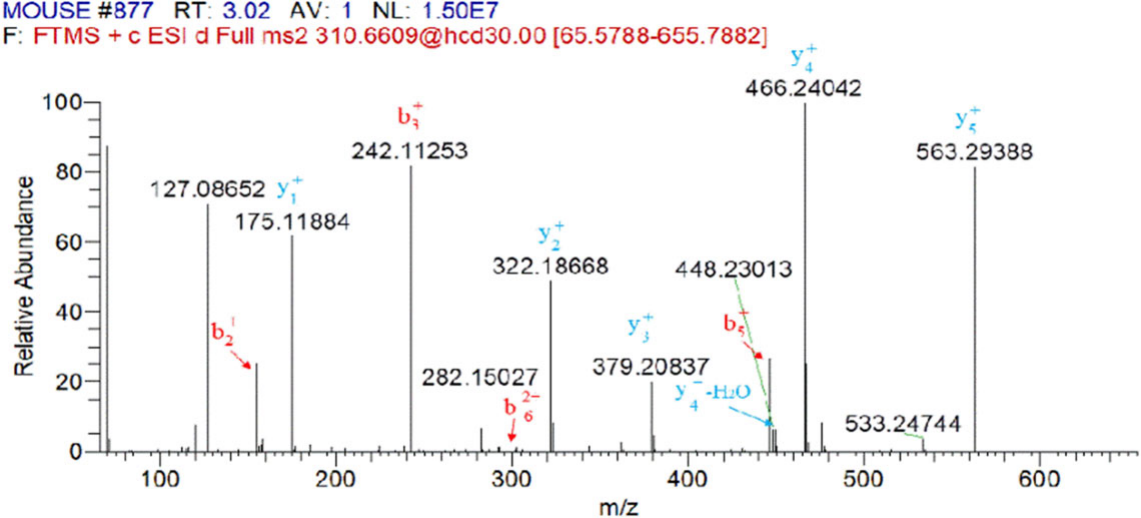
MS/MS spectrum of m/z 310.6609 detected in digested mouse skin. The MS/MS ions were searched through the SEQUEST algorithm in protein discoverer 2.4 software. The b and y ions labeled in the figure were the ions that matched the theory.

COL-I and COL-III in mouse skin contain many segments with identical amino acid sequences and some segments with differential amino acid sequences, in which the differential sequence could be used as the marker peptides. In this study, the marker peptides of COL-I and COL-III in mouse skin were identified by trypsin digestion and HPLC–MS analysis. The mass spectrometric information of collagen peptides was analyzed by the BLAST multi-sequence alignment and Protein Discoverer software. Type I and III collagen peptides, which are only detected in mouse skin, can be used as their marker peptides. Figure 1 shows total ion mass spectrum of digested mouse skin. Mass spectrometer analysis coupled with MS/MS data processing demonstrated that the digest mixture of mouse skin contained the sequence, GPSGFR. Figure 2 shows the mass spectrum of m/z 310.661 with two charges, based on the analysis of the difference in m/z of the isotope peaks by the Xcalibur software. Therefore, the molecular weight of m/z 310.6609 was 619.307 Da, which was consistent with the molecular weight of peptide (GPSGFR). The MS/MS spectrum of m/z 310.661 was shown in Fig. 3. The ions in Fig. 3 were searched through the SEQUEST algorithm in Protein Discoverer 2.4 software. The matching degree of MS/MS ions with theoretical values of the peptide (GPSGFR) was more than 75%, which indicated that the mouse collagen digestion contained the peptide (GPSGFR). The peptide (GPSGFR) could be only found in the COL-III α1 chain of mouse database, which was download from the UniProt database. Thus, it could be used as the marker peptide of COL-III in mouse skin. Eleven marker peptides of COL-I and three marker peptides of COL-III were found by the same method (Table 2).

**Table 2.** The identified marker peptides of COL-I and COL-III in mouse skin

Collagen type	Peptide chain	Sequence of marker peptide	MW	Charge	m/z
Type I	α1	GFSGLDGAK	850.4	2	426.217
		GPAGPQGPR	835.4	2	418.722
		GSEGPQGVR	885.4	2	443.722
		GETGPAGPAGPIGPAGAR	1531.8	3	511.599
	α2	GPSGPQGIR	867.4	2	434.735
		GLVGEPGPAGSK	1067.6	2	534.788
		GDQGPVGR	784.4	2	393.199
		SGQPGPVGPAGVR	1177.6	2	589.818
		PGPIGPAGPR	917.5	2	459.761
		GVVGPQGAR	839.5	2	420.738
		GPAGPSGPVGK	922.5	2	462.251
Type III	α1	GAPGPQGPR	835.4	2	418.722
		GPVGPHGPPGK	998.5	2	500.272
		GPSGFR	619.3	2	310.661

Actually, the peptides selected as marker peptides had to meet all of the following criteria at the same time. On the one hand, they had to be detected in all samples of a given collagen type. On the other hand, they had to be short peptide sequence, high matching degree, high abundance, and no hydroxylation modification [34, 35]. As shown in Table 2, the peptide (GFSGLDGAK) was one marker peptide of COL-I and had a short sequence, high matching degree and did not contain proline. Therefore, it was suitable for the quantitative analysis of COL-I. In addition to the factors (short sequence, high matching degree) consistent with the peptide (GFSGLDGAK), GPSGFR had only one proline, which was not detected to undergo any hydroxylation modification in mouse skin. Thus, GFSGLDGAK and GPSGFR were used as the marker peptides of COL-I and COL-III in mouse skin, respectively.

**Figure 4.**
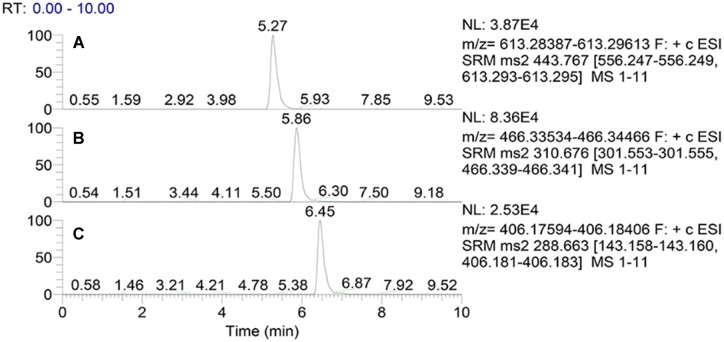
The chromatograms of marker peptide GSEGPQGVR (**A**), GPSGFR (**B**) and internal peptide GLAGMK (**C**).

To exclude the influence of matrix on quantitative results, the content of COL-I and COL-III were determined by the internal standard method. The peptide (GLAGMK) was used as the internal standard due to the fact that GLAGMK was not found in mice by Protein Information Resource and the retention time of GLAGMK was close to that of the marker peptides. The maker peptides with the concentrations from 1 to 500 μg/ml were mixed with the internal standard solution (v/v, 1:1). Figure 4 shows that the retention time of the marker peptides, GSEGPQGVR and GPSGFR, were 5.27 and 5.86 min, respectively. The retention time of the internal peptide was 6.45 min. The abscissa in this procedure was a sequence of marker peptide working solution concentrations. The ratio of the series of marker peptide working solution peak area to internal peptide peak area was used as the ordinate. The regression equation and correlation coefficient were *y* = 0.07385 + 0.000885*x*, *R*^2^ = 0.9972 (GSEGPQGVR) and *y* = 0.04585 + 0.002517*x*, *R*^2^ = 0.9995 (GPSGFR), respectively.

### Age-related change of the ratio between COL-I and COL-III

**Figure 5.**
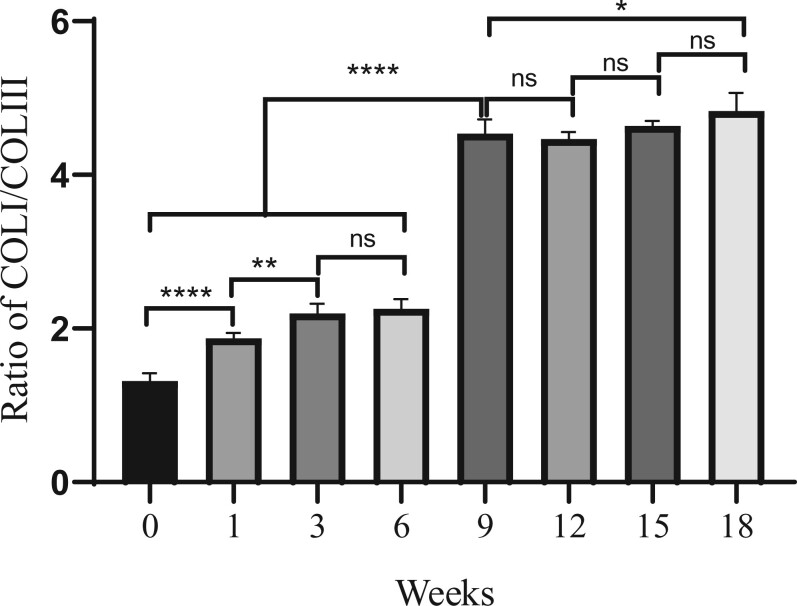
Age-related change of the COL-I/COL-III ratio in mouse skin. The COL-I/COL-III ratio was calculated by dividing the content of COL-I by COL-III. Data are shown as mean ±SDs (*n* = 6). *P < 0.05, **P < 0.01, ****P < 0.0001.

The content of COL-I and COL-III in the mouse skin was analyzed with HPLC–MS. As shown in Fig. 5, the COL-I/COL-III ratio increased first and then stabilized with aging. From the 0th to 9th week, the COL-I/COL-III ratio increased from 1.3:1 to 4.5:1, and from the 6th to 9th, the ratio increased most rapidly. It had significant differences between the 9th week and the 0–6th week. On the contrary, there was no significant difference from the 9th to 18th week, which the COL-I/COL-III ratio remained between 4.5:1 and 4.9:1. The mouse reach adulthood at the 8th or 9th week, which indicating that the COL-I/COL-III ratio in the mouse skin was in a dynamic balance after adulthood.

**Figure 6.**
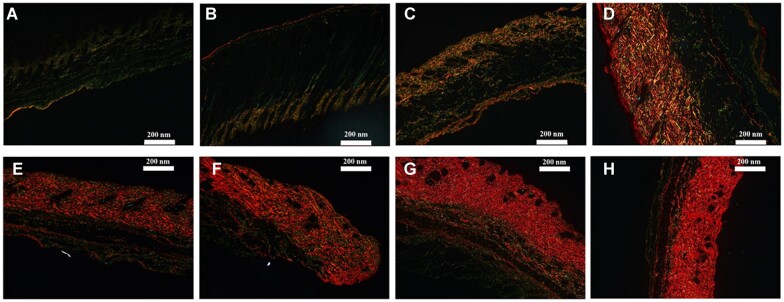
Digital images of picrosirius red staining of mouse skin viewed through polarized light microscopy. **A**, **B**, **C**, **D**, **E**, **F**, **G** and **H**: the images of picrosirius red staining at week 0, 1, 3, 6, 9, 12, 15 and 18 of mouse skin, respectively. The COL-I fibers appeared a bright red and COL-III fibers appeared yellow green.

**Figure 7.**
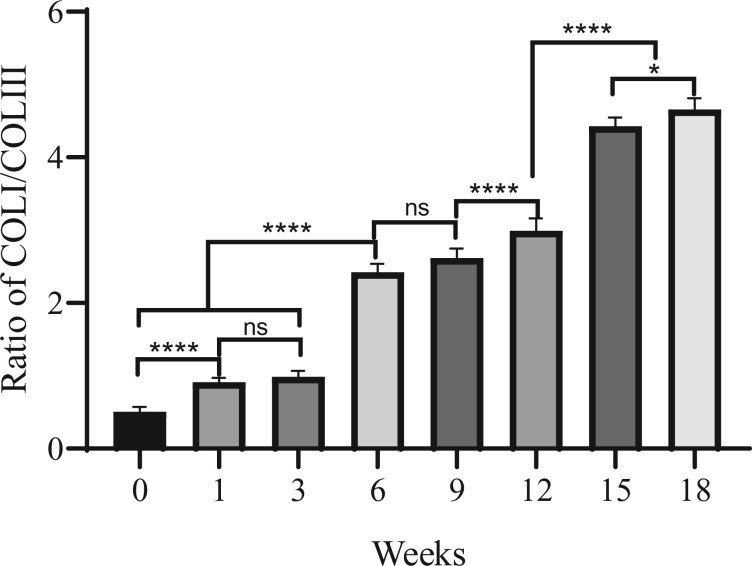
The age-related change of the COL-I/COL-III ratio in mouse skin obtained with picrosirius red staining analysis. The relative contents of COL-I and COL-III were calculated according to the color depth and area. Error bars indicate standard deviation (*n* = 6). *P < 0.05, ****P < 0.0001.

Picrosirius red staining, one of the traditional methods to analyze relative collagen content [36], was carried out to analyze COL-I and COL-III in the obtained mouse skin from the 0th to 18th week. As can be shown in Fig. 6A–H, the COL-I and COL-III were appeared red and yellow green, respectively Subsequently, the COL-I/COL-III ratio was analyzed by the IPIWIN32 software according to the color depth and area. As shown in Fig. 7, it could be observed that the COL-I/COL-III ratio in mouse skin increased with aging, in which the ratio increased significantly from the 3rd to 6th week and the 12rd to 15th week (*P* < 0.0001). From the 6th to 12th week, the ratio increased between 2.4:1 and 3.0:1 (*P* < 0.0001). The ratio was no significant differences between the 15th week and the 18th week, which was from 4.3:1 to 4.7:1. It can be confirmed that the total variation trend of the ratio obtained by picrosirius red staining was consistent with the HPLC–MS method.

### Age-related change of total collagen

**Figure 8.**
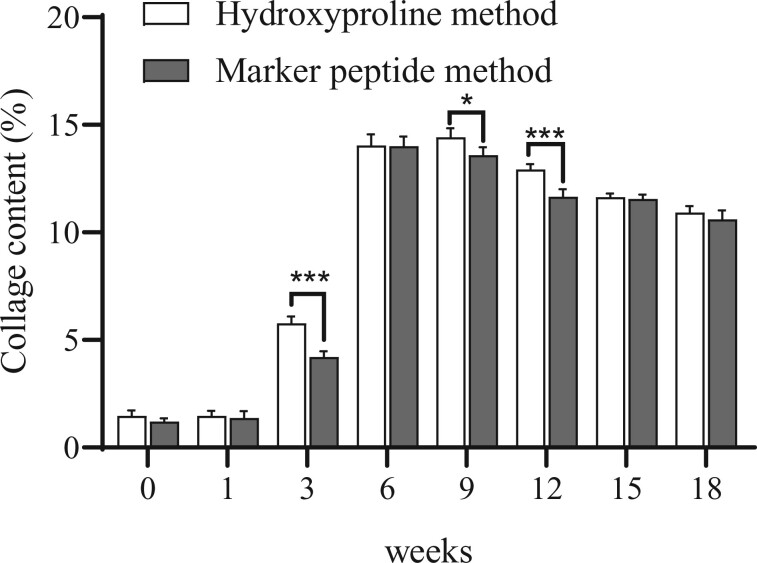
The age-related change of total collagen content in mouse skin. Hydroxyproline is the specific amino acid of collagen. In the hydroxyproline method, the total collagen content was obtained with dividing the hydroxyproline content by the hydroxylation ratio. In the marker peptide method, the total collagen content was the content of COL-I and COL-III. Error bars indicate standard deviation (*n* = 6). *P < 0.05, ***P < 0.001.

To verify the accuracy of the HPLC**–**MS method, the content of total collagen in mouse skin was determined by the hydroxyproline method. As shown in Fig. 8, the age-related change of the total collagen content in the mouse skin obtained by the hydroxyproline method was slightly higher than the total amount of COL-I and COL-III obtained by the HPLC**–**MS method. It indicated that there was no significant difference between the two methods except at the 3th and 12th week.

It can be observed that the total collagen content obtained by the two different methods in mouse skin both increased rapidly from the 0th to 6th week. In the 0th and the 1st week the total collagen content was about 1.2–1.5% in the mouse skin. It increased to 14% in the 6th week. The content was stable for 14–14.5% from the 6th to 9th week. After the 9th week, the total collagen content decreased slowly, and by the 18th week, it dropped to 10.6%.

### Age-related change of collagen fibril diameter

**Figure 9.**
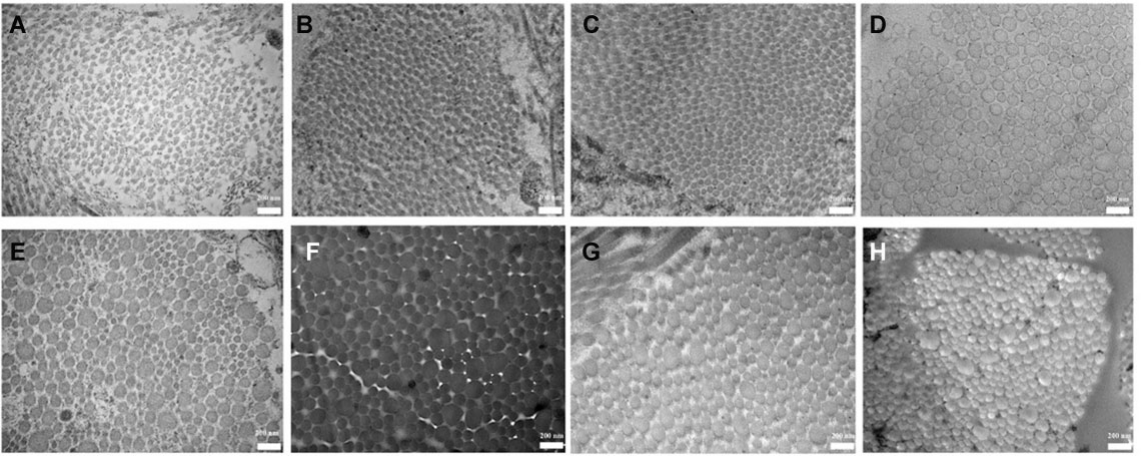
The fibril diameter of mouse skin from the 0th to the 18th week. **A**, **B**, **C**, **D**, **E**, **F**, **G** and **H**: the fibril of diameter images of mouse skin at week 0, 1, 3, 6, 9, 12, 15 and 18, respectively.

**Figure 10.**
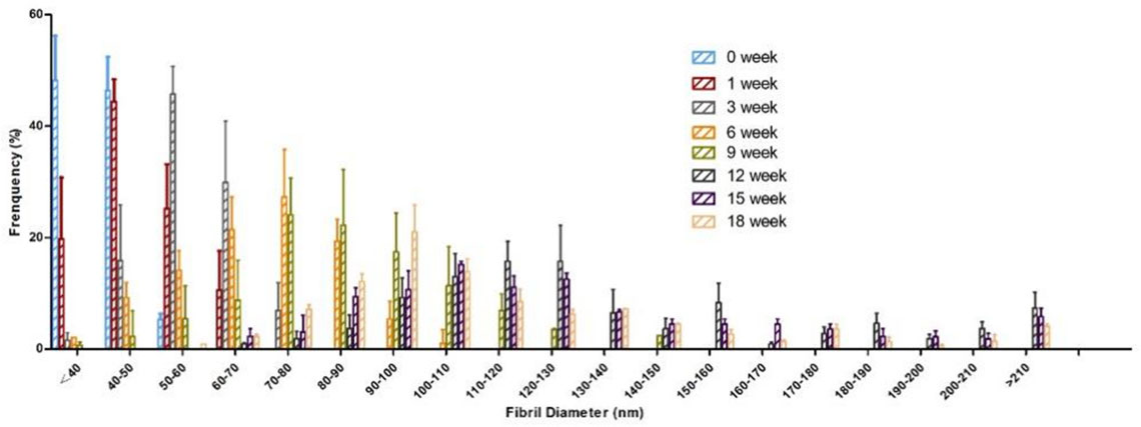
The fibril diameter distribution of mouse skin. The frequency (%) of collagen fibrils in the skin, with a given diameter (nm), shows a significant difference in distribution with different weeks.

**Figure 11.**
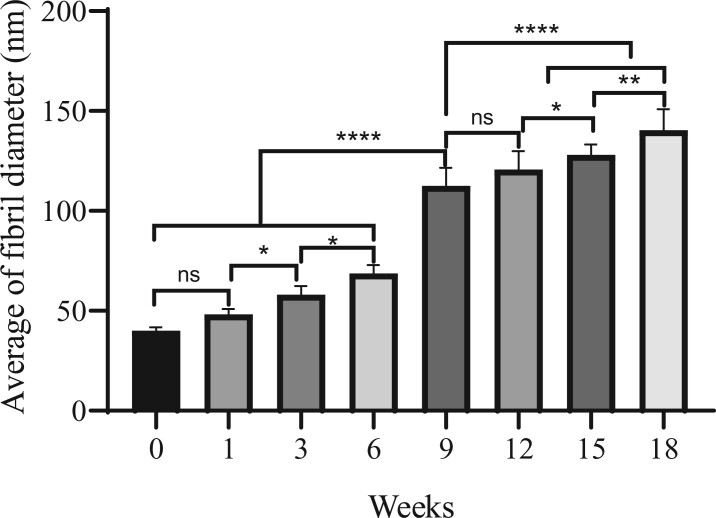
The age-related change of the average fibril diameter in mouse skin. *P < 0.05, **P < 0.01, ****P < 0.0001.

To determine the relationship between the COL-I/COL-III ratio and collagen fibril diameter, the mouse skin collagen fibril diameters from the 0th to 18th week were analyzed in this study (Fig. 9). The fibril diameters were obtained with TEM and calculated by Image J software. In Fig. 10, the diameter distribution of collagen fibril was relatively narrow from the 0 to 6th week, but it showed uneven and wider distribution with aging after the 6th week. Figure 11 presents the age-related change of the average fibril diameter in mouse skin. The results revealed that the average collagen fibril diameter increased gradually with aging. The mean fibril diameter significantly increased from 40 to 69 nm between the 0th to 6th week (*P* < 0.0001). By the 9th week, it dramatically increased to 112 nm (*P* < 0.0001). There was no marked increase from the 9th to the 12th week (*P* > 0.05). Then it showed a slowly increasing trend from 120 to 130 nm from the 12th to the 15th week (*P* < 0.05). At last, the fibril diameter increased to 140 nm at the 18th week, which was a very noticeable increase from the 9th week (*P* < 0.0001).

## Discussion

COL-I and COL-III are fibrillar collagen, and mainly located in the dermis [37]. They are associated in the skin and form fibrils with a characteristic 67 nm periodicity [38]. They play the important role in maintaining the mechanical properties of the skin [19]. The COL-I/COL-III ratio is closely related to the healthy status of the skin and the fibril diameter [26]. The method for quantification of collagen based on LC–MS and marker peptides was established. The COL-I/COL-III ratio increased first and then stabilized with aging. In the 0th week, the COL-I/COL-III ratio was only 1.3:1. Whereas, the COL-I/COL-III ratio increased to 4.5–4.9:1 from the 9th to 18th week. The total content of COL-I and COL-III was increased from the 0th to 9th week and then decreased. The reason for these changes is that the relative content of COL-I increased from the 0th to 18th, while the relative content of COL-III decreased with aging. This is consistent with the fact that skin becomes less elastic and wrinkles increase with aging [37]. Furthermore, the picrosirius red staining was used to perform this phenomenon. The variation trend of the COL-I/COL-III ratio with aging obtained by picrosirius red staining was consistent with the HPLC**–**MS method. The reason for the minor differences may be that the picrosirius red staining was quantified by the area and the depth of the color. It is a semi-quantitative method, and there may be some micro-error in the analysis results.

The total collagen content in the mouse skin obtained by the hydroxyproline method was slightly higher than the total amount of COL-I and COL-III obtained by the marker peptide method. This is mainly because there are some other types of collagens, such as Type V, VII and XII collagens, in the mouse skin [39, 40]. This verified that the HPLC**–**MS method was reliable in analyzing the content of collagen. And the total content of collagen obtained by the two different methods in mouse skin both increased rapidly before the 6th week. This mainly because that the synthetic rate was active before the adulthood. After the 9th week, the total content of collagen was decreased slowly. The main reason was that the synthetic rate of the collagen was still active in the skin. Whereas the degradation rate of collagen in the mouse skin was greater than the synthetic rate in the adulthood [17].

The diameter of collagen fibril increased with aging and was proportionally to the COL-I/COL-III ratio. This indicated that the COL-I/COL-III ratio was the main factor in affecting the diameter of collagen fibril [11, 16]. The main reason was that COL-I and COL-III had different functions during fibrillogenesis. COL-I as the ‘building unit’ of the fibril was in the center of the fibril. While COL-III which located in the periphery of the fibril had a greater effect on fiber diameter, such as regulation of fibril growth, control of fibril diameter and interactions with other proteins [41, 42]. Furthermore, the diameter of collagen fibril showed uneven distribution in the adulthood. This may due to that the collagen fibril was intact, tightly packed, well-organized in the young skin. However, the collagen fibril was fragmented, disorganized and sparse in the aged skin [43]. And COL-III tends to form finer fibrils, arranged in narrower bundles of fibrils than COL-I [37]. Collagen fibrils are short and narrow in the embryo. The growth of the collagen fibril diameter was to match the changing mechanical requirements [44].

## Conclusion

In this study, we firstly established a quantitative method of COL-I and COL-III based on HPLC**–**MS. Afterwards, the content of COL-I and COL-III were quantified in the mouse skin of different weeks, in which the COL-I/COL-III ratio and the content of the total collagen with aging was calculated. Furthermore, the diameter of collagen fibril in mouse skin was analyzed. It was found that the COL-I/COL-III ratio was the main factor, which affecting the diameter of collagen fibril. The content of the total collagen increased first and then decreased with aging. This study was a systematic study on the COL-I/COL-III ratio and collagen fibril diameter with aging. We believe it can provide the theoretical basis for skin aging and diseases related to collagen.
